# Short-term outcomes of Ivor Lewis vs. McKeown esophagectomy: A meta-analysis

**DOI:** 10.3389/fsurg.2022.950108

**Published:** 2022-10-28

**Authors:** Huajie Xing, Mengyu Hu, Zhiqiang Wang, Yuequan Jiang

**Affiliations:** ^1^ Department of Thoracic Oncology, Chongqing University Cancer Hospital, Chongqing, China; ^2^ Department of Radiation Oncology, Chongqing University Cancer Hospital, Chongqing, China

**Keywords:** esophagectomy, McKeown, Ivor Lewis, anastomosis leak, meta-analysis

## Abstract

**Objective:**

The objective of this article is to assess the rate of anastomotic leak and other perioperative outcomes in patients undergoing esophagectomy with either thoracic or cervical anastomosis.

**Methods:**

This meta-analysis was conducted by searching relevant literature studies in Web of Science, Cochrane Library, PubMed, and Embase databases. Articles that included patients undergoing esophagectomy and compared perioperative outcomes of McKeown with Ivor Lewis procedures were included. The primary outcome parameter was anastomotic leak, and secondary outcome parameters were grade ≥2 anastomotic leak, chylothorax, recurrent laryngeal nerve injury, hospital length of stay, intensive care unit (ICU) length of stay, postoperative mortality rate, operative time, blood loss, R0 resection rate, and lymph nodes examined.

**Results:**

A total of eight studies, with 3,291 patients (1,857 Ivor Lewis procedure and 1,434 McKeown procedure) were eligible for analysis. Meta-analysis showed that Ivor Lewis procedure was associated with lower rate of anastomosis leak of all grades [risk ratio (RR), 0.67; 95% confidence interval (CI), 0.55–0.82; *P* = 0.0001], lower rate of recurrent laryngeal nerve injury (RR, 0.14; 95% CI, 0.08–0.25), and shorter length of hospital stay (weighted mean difference, 0.13; 95% CI, 0.04–0.22). Grade ≥2 anastomotic leak, chylothorax, ICU length of stay, postoperative mortality rate, operative time, blood loss, R0 resection rate, and lymph nodes examined were similar between the two groups.

**Conclusions:**

Although all grades of anastomotic leak and recurrent laryngeal nerve injury are higher in the McKeown procedure, this meta-analysis supports similar short-term outcomes and oncological efficacy between Ivor Lewis and McKeown esophagectomy.

## Introduction

Esophagectomy is considered the cornerstone of curative treatment for esophageal cancer, which is the sixth cause of cancer-associated deaths worldwide (1). Esophagectomy can be performed in the transthoracic or the transhiatal manner. Transthoracic esophagectomy, which could be performed with either intrathoracic anastomosis (the Ivor Lewis procedure) or cervical anastomosis (the McKeown procedure), is favored by many surgeons because it allows for adequate thoracic lymph node dissection.

Despite the prolonged life expectancy, esophagectomy has been plagued by high rates of morbidity and mortality. Although the mortality rate of esophagectomy has significantly decreased in the last three decades, this operation still carries a high risk of death compared with most surgically treated cancers, and postoperative complications continue to range from 26% to 41% (2, 3). Postoperative complications are directly linked to many important outcomes including mortality rate, length of hospital stay, costs, readmission rate, early cancer recurrence, survival, and quality of life (4–6).

Anastomotic leak refers to full thickness gastrointestinal defects involving esophagus, anastomosis, staple line, or conduit, and it contributes to a marked increase in morbidity and mortality rates after esophagectomy (7). According to the study conducted by Chidi et al., patients who experienced an anastomotic leak have a sixfold increase in mortality rate compared with those without leak (8). There is conflicting evidence about the factors that contribute to anastomotic leak. Prior studies have compared anastomotic leak rates after cervical and thoracic esophagogastric anastomosis. Although some studies have shown lower anastomotic leak rates after intrathoracic anastomosis (9–13), others failed to display a difference (8, 9, 14–16).

While the debate over the superiority of either approach (Ivor Lewis vs. McKeown) continues, we sought to explore this problem with a systemic review and meta-analysis. The aim of this study was to compare transthoracic esophagectomy by intrathoracic anastomosis with transthoracic esophagectomy by cervical anastomosis, in terms of anastomotic leak and other postoperative morbidity and mortality outcomes in patients with potentially curable middle to distal esophageal or gastroesophageal junction cancer.

## Materials and methods

### Literature research

All procedures of this meta-analysis were guided by Cochrane Handbook for Systematic Reviews of Interventions Version 5.1.0, and was reported based on the Preferred Reporting Items for Systematic Reviews and Meta-Analyses (PRISMA) Statement. A literature search of the Web of Science, Cochrane Library, PubMed, and Embase databases was performed by two independent researchers (HX and YJ). Studies published from 2010 to October 2021 were included. To perform a comprehensive search, the following keywords and MeSH terms were used in different patterns: “esophagus cancer”, “esophagectomy”, “Minimally Invasive esophagectomy”, “Ivor-Lewis”, and “Mckeown”. The reference lists of the included literature studies were screened again in order to identify potentially relevant articles.

### Inclusion and exclusion criteria

All records were screened on the basis of title and abstract by two authors (MH and HX) independently. Full text of the studies that were not excluded in the screening stage was further assessed for eligibility. If discrepancies occurred, discussion with a third author (ZW) was held to reach a consensus. The following criteria were used for study inclusion: randomized controlled trials (RCTs) or cohort studies that compared perioperative outcomes of Ivor Lewis vs. McKeown procedures in middle to distal thoracic esophageal or junctional cancer patients; sufficient perioperative outcome data could be obtained; and the most recent or complete study if based on overlapping patients. The exclusion criteria are as follows: papers without relevant data for analysis; description of one surgical technique only; consisted of less than 10 patients or fewer than 10% of total enrolment in either arm; papers that were not published in English; and commentaries, case reports, abstracts, conference reports, reviews, letters, and experiments.

### Data extraction and quality assessment

The primary outcome of this study is all grades of anastomotic leak. The secondary outcomes include grade ≥2 anastomotic leak, 30- and 90-day mortality rates, recurrent laryngeal nerve (RLN) injury, chylothorax, operation time, blood loss, R0 resection rate, number of retrieved lymph node, length of intensive care unit (ICU), and hospital stay. The following data were extracted from articles by two investigators (MH and HX) in a standardized form, including the publication details, study design, patient characteristics, duration of the study, country, number of patients included, surgical procedures, and postoperative outcomes. Any discrepancies were judged by a third author (ZW) to reach consensus.

### Assessment of bias

The quality assessment of cohort studies was evaluated using the Newcastle–Ottawa Scale (NOS) (http://www.ohri.ca/programs/clinical_epidemiology/oxford.asp) that contains three sections, namely, the selection of the involved groups, the comparability between the groups, and the assessment of follow-up and outcomes. The number of total stars was recorded to reflect the quality of the included studies, which ranged from 1 to 9. Studies with a score of 7 or greater, 6 or 5, and 4 or less were determined to be at a low, medium, and high risk of bias, respectively. The Jadad scale was used to assess the quality of randomized trials (17). Publication bias was assessed by funnel plot and Egger's test.

### Statistical analysis

Statistical analyses were conducted using Review Manager 5.3 (Nordic Cochrane Centre, Cochrane Collaboration). Overall incidence rates (odds ratios)/weighted mean difference with 95% confidence intervals (CIs) were calculated for binary and continuous parameters, respectively. Forest graphs were applied to present the meta-analysis results. The statistical heterogeneity of the included literature studies was assessed by *I*^2^ statistic. *I*^2^ values ≤50%, 50%–74%, and ≥75% indicate low, moderate, and high heterogeneity, respectively. A fixed-effects model was chosen to perform the meta-analysis when the *I*^2^ value was ≤50%. A random-effects model was used when the *I*^2^ value was >50%. Statistical significance value was set at *P* < 0.05.

## Results

### Study characteristics

**Figure 1:**
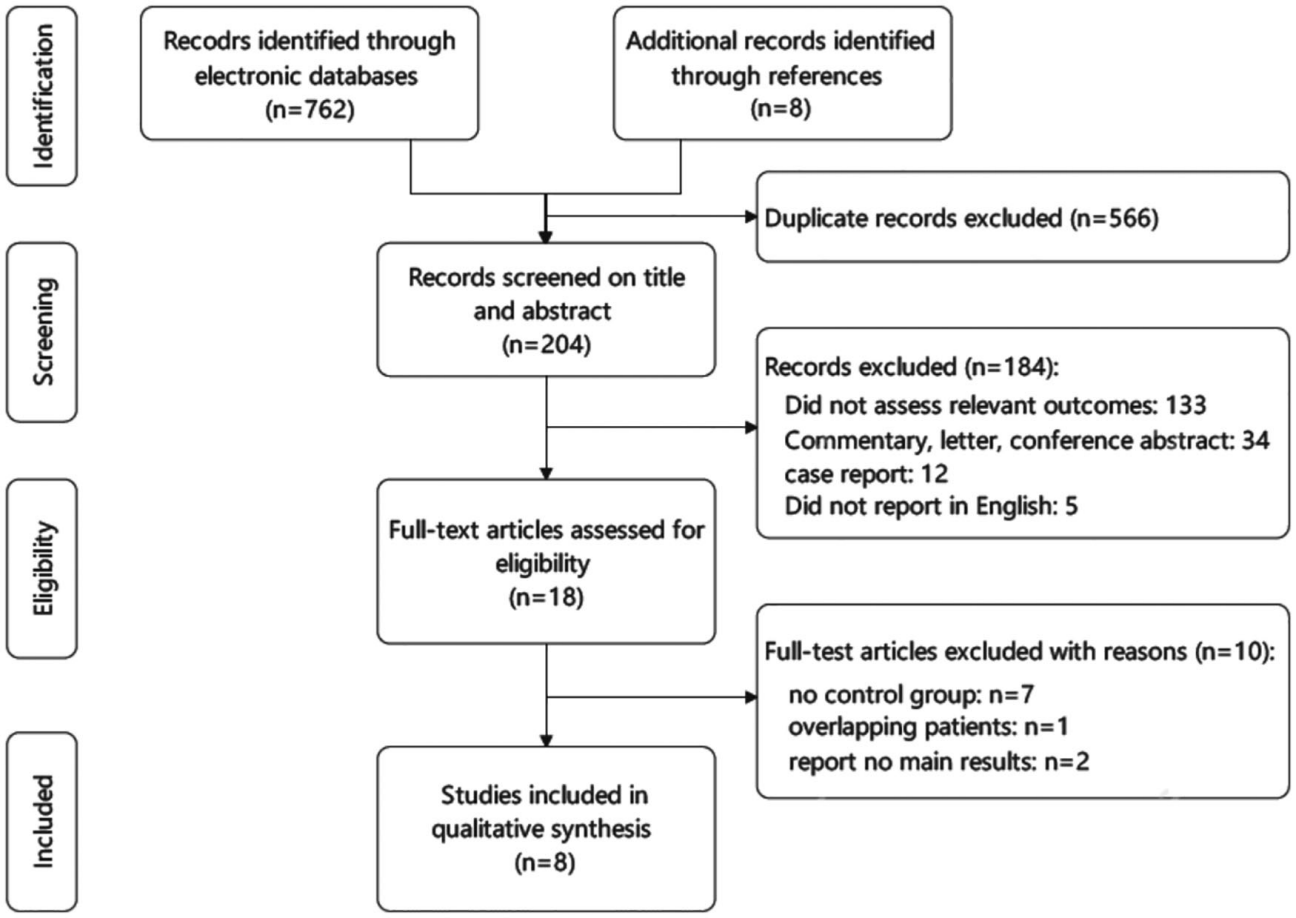
Preferred Reporting Items for Systematic Reviews and Meta-Analyses flow diagram.

A flow diagram of the literature search is illustrated in Figure 1. With our searching strategy, eight studies were finally subjected to this meta-analysis (8–11, 13–16). The selected eight studies were published from March 2012 through 2021. One study was a randomized clinical trial, and the remaining seven were retrospective cohort studies (the NOS scores of the seven studies were 7 or 8). A total of 3,291 patients were included, of whom 1,857 (56.4%) received the Ivor Lewis procedure and 1,434 (43.6%) received the McKeown procedure. Totally, 311 (9.5%) patients in two studies received open esophagectomy, while the remaining patients received total or hybrid minimal invasive esophagectomy. Characteristics and NOS quality star of the included studies are summarized in Table 1.

**Table 1:** Characteristics of included studies.

Study	Year of publication	Country	Sample size		Study type	Tumor location	Surgery type	Anastomosis details	NOS
			IL	M					
Zhai et al.	2015	China	32	40	Retrospective	Middle/lower	MIE	Mechanical	7
Liu et al.	2018	China	152	44	Retrospective	Lower	MIE (42.8%)/OE	Mechanical/handsewn	7
Van Workum et al.	2021	Netherlands	130	132	RCT	Middle/lower	MIE/HMIE	Mechanical/handsewn	NA
Brown et al.	2017	United States	49	61	Retrospective	NA	MIE	Mechanical	8
Luketich et al.	2012	United States	530	481	Retrospective	NA	MIE	Mechanical/handsewn	7
Chidi et al.	2020	United States	351	270	Retrospective	NA	MIE (68%)/OE	NA	7
Schmidt et al.	2017	Switzerland	188	146	Retrospective	Middle/lower	MIE	NA	8
Shi et al.	2021	China	68	68	Retrospective	Middle/lower	MIE	Mechanical	7

NOS, Newcastle–Ottawa Scale; RCT, randomized clinical trial; MIE, minimally invasive esophagectomy; HMIE, hybrid minimally invasive esophagectomy; OE, open esophagectomy; NA, not available; IL, Ivor Lewis; M, McKeown.

### Primary outcome

**Figure 2:**
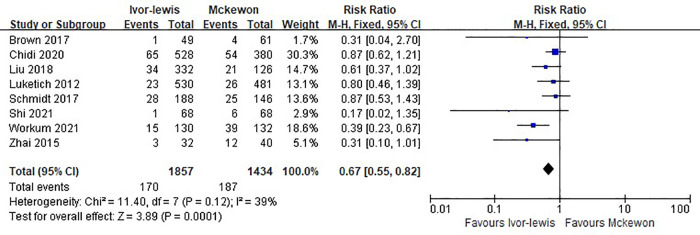
Forest plots of the all grades anastomotic leak analysis in Ivor Lewis procedure vs. McKeown procedure.

All included studies were eligible for the anastomotic leak analysis. The Ivor Lewis procedure had a lower rate of all grades of anastomotic leak compared with the McKeown procedure [risk ratio (RR), 0.67; 95% CI, 0.55–0.82; *P* = 0.0001; *I*^2^ = 39%] (Figure 2).

### Secondary outcomes

**Figure 3:**
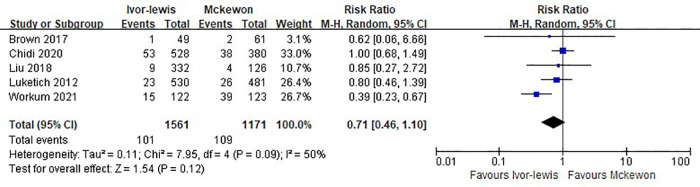
Forest plots of the grade ≥2 anastomotic leak analysis in Ivor Lewis procedure vs. McKeown procedure.

Pooled effects for the secondary outcomes are shown in Table 2. Five studies evaluated grade ≥2 anastomotic leak, and there was no significant difference between the Ivor Lewis and McKeown procedure (RR = 0.71, 95% CI, 0.46–1.10, *P* = 0.09) (Figure 3).

**Table 2:** Secondary outcomes.

Outcome	Number of studies	Total number of patients	*I* ^2^	Effect	RR or WMD	*P*-value
Operation time	5	1,793	54%	FE	−5.14 (−11.84 to 1.57)	0.13
Blood loss	4	885	56%	RE	−1.25 (−18.34 to 15.84)	0.89
Number of lymph nodes	6	2,179	88%	RE	−0.70 (−3.53 to 2.13)	0.63
R0 rate	6	3,015	0%	FE	1.01 (0.99 to 1.02)	0.28
Length of ICU stay	4	2,048	91%	RE	0.25 (−0.41 to 0.91)	0.46
Length of hospital stay	6	2,015	0%	FE	−0.69 (−1.18 to −0.19)	0.006
RLN injury	6	2,032	0%	FE	0.14 (0.08 to 0.25)	<0.0001
Chylothorax	5	1,021	0%	FE	0.91 (0.54 to 1.54)	0.74

ICU, intensive care unit; RLN, recurrent laryngeal nerve; FE, fixed effect; RE, randomized effect; RR, risk ratio; WMD, weighed mean difference.

**Figure 4:**
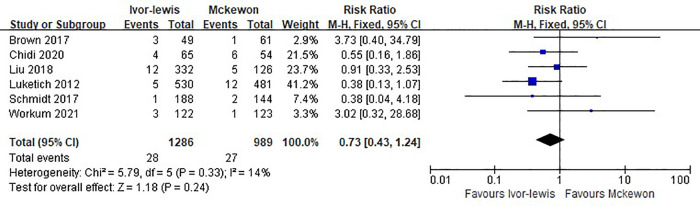
Forest plots of the 30-day mortality analysis in Ivor Lewis procedure vs. McKeown procedure.

Six studies involving 2,275 cases reported the 30-day mortality rate, with 2.2% (28 in 1286) for the Ivor Lewis procedure and 2.7% (27 in 989) for the McKeown procedure. No statistically significant difference was found between the two groups (RR = 0.73, 95% CI, 0.43–1.24, *P* = 0.24) (Figure 4).

**Figure 5:**
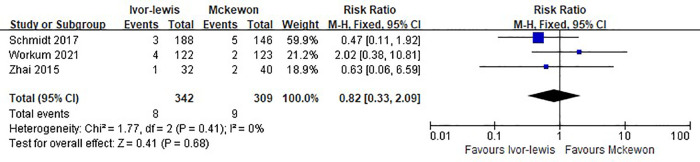
Forest plots of the 90-day mortality analysis in Ivor Lewis procedure vs. McKeown procedure.

Three studies involving 651 cases reported the 90-day mortality rate, and there was no significant difference between the two procedures (RR = 0.82, 95% CI, 0.33–2.09, *P* = 0.68) (Figure 5).

Intraoperative data were also pooled and the results are shown in Table 2. The Ivor Lewis procedure was comparable with the McKeown procedure in terms of operation time, blood loss, lymph nodes resected, and R0 resection rate. Length of hospital stay was 0.69-days shorter in the Ivor Lewis group, while the length of ICU stay was comparable. The McKeown procedure is associated with increased risk of recurrent laryngeal nerve injury, while the incidence of chylothorax between the two procedures was similar.

### Subgroup analysis

Subgroup analysis was conducted in studies that only contain minimally invasive esophagectomy cases. Five studies, with a total of 1,663 cases were included in the subgroup analysis. The pooled estimate favored the Ivor Lewis procedure in terms of anastomotic leak, with an RR of 0.68 (95% CI, 0.49–0.96; *P* = 0.03; *I*^2^ = 23%).

### Publication bias analysis

Publication bias was assessed by Egger's test, and significant statistical publication bias was detected with anastomotic leak. The trim-and-fill computation was carried out to estimate the effects of publication bias on the results, which indicated that the results were consistent and stable.

## Discussion

As one of the most common and severe complications after esophagectomy, anastomotic leak is associated with considerable morbidity, decreased quality of life, a mortality rate of 2%–12%, and decreased long-term survival (18). The incidence of anastomotic leak varies in different studies, which can be up to 31.7% (10). Transthoracic esophagectomy can be performed with either intrathoracic or cervical anastomosis. For a long time, the relationship between type of surgery and leak rate has been a question of debate.

In the present meta-analysis, the Ivor Lewis procedure was associated with a significantly lower incidence of anastomotic leak compared with the McKeown procedure. There were several possible explanations for the difference in the anastomotic leak rate. First, blood supply is always thought to be the key factor affecting wound healing. For esophagectomy with intrathoracic anastomosis, relatively less ischemia at the tip of the shorter gastric tube may lead to lower anastomotic leak rate. Moreover, compression of the gastric tube by the thoracic outlet may also result in poorly vascularized anastomosis in the neck (11). In addition to blood supply, relatively high tension of the cervical anastomosis site may be another potential risk factor for leak. Moreover, leak in the neck is easier to be found compared with intrathoracic. Redness and purulence of the neck skin, early signs of leak, are easy to be found by inspection and could lead to further investigation to confirm the diagnosis, which could lead to significant bias between the two groups. In the multicenter randomized trial by van Workum et al. (10), they employed strict definition and classification for anastomotic leak. The results showed that total and severe leak rates were both lower in the intrathoracic group.

In addition to the incidence of anastomotic leak, it is also important to appreciate the severity of anastomotic leak. According to the Esophagectomy Complications Consensus Group (ECCG), the severity of anastomotic leak could be divided into three grades. Grade I means leak requiring no change in therapy or treated medically or with dietary modification, while grade II and grade III need nonsurgical or surgical reintervention (6). This study revealed that the difference in anastomotic leak requiring reintervention or reoperation (grade II and grade III) was not significant between the two groups, probably because cervical leakage is easier to treat to prevent the deterioration of the leak condition, despite higher rates in the McKeown group. On the contrary, once the intrathoracic leak occurs, it is much more difficult to deal with. Many surgeons hold the view that intrathoracic anastomotic leakage is more severe than cervical anastomotic leakage, although the evidence is scarce (19). Therefore, surgeons may prefer to take more aggressive measures for intrathoracic anastomotic leak, which could partially explain the similar grade II and grade III leak rates in the intrathoracic group, while the total leak rate is lower. According to the study by Linden et al., severe anastomotic leak was associated with 1.5-fold postoperation mortality rate after esophagectomy, while leak requiring no intervention or medical reintervention had no impacts (20). This is consistent with our finding that the 30- and 90-day mortality rates between the two surgery groups was not statistically significant. This result is also supported by other studies (11, 21). Recently some studies reported a lower 30-day mortality rate in the Ivor Lewis procedure (12). This may be a result of learning curve. Most centers adopted the Ivor Lewis procedure after the McKeown procedure, thus leading to a relatively higher mortality rate in early-stage Ivor Lewis procedure. Median ICU length of stay was similar between the McKeown and Ivor Lewis esophagectomy. Less recurrent laryngeal nerve injury was found in the Ivor Lewis procedure, which may be the result of the omitted third incision in the left neck. Hospital length of stay was longer in the McKeown group. This could be a result of less severe complications, which is proved by some other studies (10), but a 0.69 day mean difference is unlikely to be clinically significant. Operation time and blood loss were also similar between the two groups. Taken together, we point out that the two procedures are comparable for surgical safety.

Oncologic efficacy was evaluated in this review. Although the McKeown procedure has the advantage of cervical lymph node dissection and more proximal resection margin, no difference in total lymph nodes retrieved during surgery and R0 resection rate between the two procedures was detected. This could partially be explained that all included studies in this meta-analysis enrolled middle to distal thoracic esophageal or gastroesophageal junction cancer patients, in whom cervical lymph node dissection is not required and both procedures are oncological feasible. According to the study by Lagergren et al., the extent of lymphadenectomy during surgery for esophageal cancer might not influence 5-year all-cause or disease-specific survival (22). On the other hand, a randomized clinical trial also proved that there was no improvement in overall survival or disease-free survival after esophagectomy with three-field lymphadenectomy over two-field lymphadenectomy for middle and lower thoracic esophageal cancer (23). Long-term survival result was unavailable from this study. According to a multicenter observational study from China, minimally invasive McKeown esophagectomy was associated with improved overall survival and a decreased risk of disease recurrence compared with the Ivor Lewis procedure (24). However, related evidence is still insufficient, and more high-quality studies are needed to compare the long-term survival of the two procedures.

Overall, a marked difference in favor of the Ivor Lewis procedure in terms of all-grade anastomotic leaks and RLN injury was identified, while severe anastomotic leak, 30-d and 90-day mortality rates, and oncological efficacy were similar between the two procedures based on the available evidence. Potential advantages of the McKeown esophagectomy include a less technically challenging anastomosis procedure, and if an anastomotic leak occurs, it can be managed more easily than an intrathoracic leak. Additionally, McKeown esophagectomy is suitable for tumor above the carina. Taken together, we point out that the two procedures are of equal perioperative and oncological safety, and are both acceptable when clinically and oncologically appropriate. Based on this information, surgeons should continue to evaluate the benefits and risks of each surgical approach for individual patients. Surgeon experience and patient risk factors should ultimately determine which approach is ideal.

We noticed that there are two meta-analyses on this topic (25, 26). However, the conclusion from these two studies was controversial. In the study by Deng et al., cases were included as early as 1998, when minimal invasive esophagectomy had just started. The leakage rate could be significantly higher due to the existence of a learning curve, which had been proved (27). Moreover, some benign esophageal disease cases were also included (28). In the meta-analysis by van Workum et al., only five retrospective studies were included. The major strength of our study is that only studies published in the last decade were included, and in this way we could minimize the influence of learning curve. After careful selection, eight studies were eligible, including one randomized clinical trial. The strict inclusion criteria ensured a high-quality meta-analysis.

Some limitations should also be discussed. For a long time, there was no clear and universal definition and classification of anastomosis leak, which caused reporting bias among different studies. In 2015, the ECCG proposed standardized definitions for complications after esophagectomy, and hopefully this will lead to more uniform reporting in future studies. Second, limited information about additional factors that may influence the leakage rate could be obtained and analyzed in this study, including preoperative nutrition status, functional status, hospital or surgeon volume, neoadjuvant treatment, anastomotic technique (handsewn vs. stapled), use of ischemic preconditioning, and certain comorbidities. Thus, the comparability of the two groups could not be fully assessed.

## Conclusion

In conclusion, this meta-analysis supports similar short-term outcomes and oncological efficacy between Ivor Lewis and McKeown esophagectomy, though all-grade anastomotic leak and RLN injury are higher in the McKeown procedure. Data on long-term survival, quality of life, and cost-effectiveness are needed to fully justify a preferred esophagectomy technique.

## Data Availability

The raw data supporting the conclusions of this article will be made available by the authors, without undue reservation.
